# A 3D atlas of the human developing pancreas to explore progenitor proliferation and differentiation

**DOI:** 10.1007/s00125-024-06143-2

**Published:** 2024-04-17

**Authors:** Adrian Villalba, Yorick Gitton, Megumi Inoue, Virginie Aiello, Raphaël Blain, Maryne Toupin, Séverine Mazaud-Guittot, Latif Rachdi, Henrik Semb, Alain Chédotal, Raphaël Scharfmann

**Affiliations:** 1 Institut Cochin, CNRS, Inserm, Université Paris Cité, Paris, France; 2 Inserm, CNRS, Institut de la Vision, Sorbonne Université, Paris, France; 3 Inserm, EHESP, IRSET (Institut de Recherche en Santé, Environnement et Travail), UMR_S 1085, Université Rennes, Rennes, France; 4 Institute of Translational Stem Cell Research, Helmholtz Diabetes Center, Helmholtz Zentrum München, München, Germany; 5 Institut de pathologie, groupe hospitalier Est, hospices civils de Lyon, Lyon, France; 6 MeLiS, CNRS UMR5284, Inserm U1314, University Claude Bernard Lyon 1, Lyon, France

**Keywords:** Human fetal pancreas, Insulin-producing cells, Light-sheet fluorescence microscopy, Pancreatic progenitors, PDGF signalling, Proliferating cells, Type 1 diabetes

## Abstract

**Aims/hypothesis:**

Rodent pancreas development has been described in great detail. On the other hand, there are still gaps in our understanding of the developmental trajectories of pancreatic cells during human ontogenesis. Here, our aim was to map the spatial and chronological dynamics of human pancreatic cell differentiation and proliferation by using 3D imaging of cleared human embryonic and fetal pancreases.

**Methods:**

We combined tissue clearing with light-sheet fluorescence imaging in human embryonic and fetal pancreases during the first trimester of pregnancy. In addition, we validated an explant culture system enabling in vitro proliferation of pancreatic progenitors to determine the mitogenic effect of candidate molecules.

**Results:**

We detected the first insulin-positive cells as early as five post-conceptional weeks, two weeks earlier than previously observed. We observed few insulin-positive clusters at five post-conceptional weeks (mean ± SD 9.25±5.65) with a sharp increase to 11 post-conceptional weeks (4307±152.34). We identified a central niche as the location of onset of the earliest insulin cell production and detected extra-pancreatic loci within the adjacent developing gut. Conversely, proliferating pancreatic progenitors were located in the periphery of the epithelium, suggesting the existence of two separated pancreatic niches for differentiation and proliferation. Additionally, we observed that the proliferation ratio of progenitors ranged between 20% and 30%, while for insulin-positive cells it was 1%. We next unveiled a mitogenic effect of the platelet-derived growth factor AA isoform (PDGFAA) in progenitors acting through the pancreatic mesenchyme by increasing threefold the number of proliferating progenitors.

**Conclusions/interpretation:**

This work presents a first 3D atlas of the human developing pancreas, charting both endocrine and proliferating cells across early development.

**Graphical Abstract:**

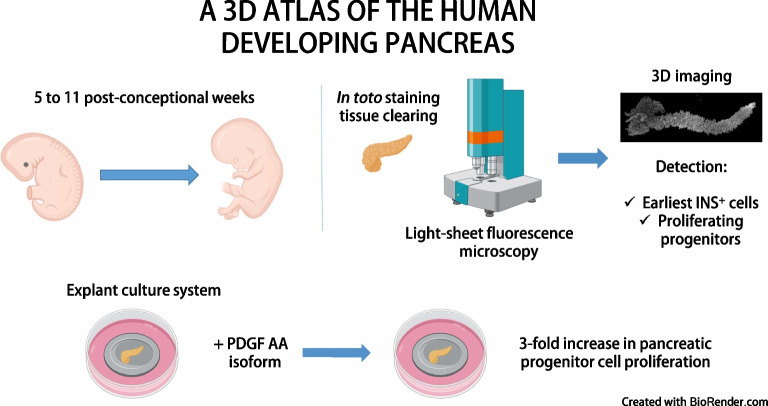

**Supplementary Information:**

The online version contains peer-reviewed but unedited supplementary material available at 10.1007/s00125-024-06143-2.


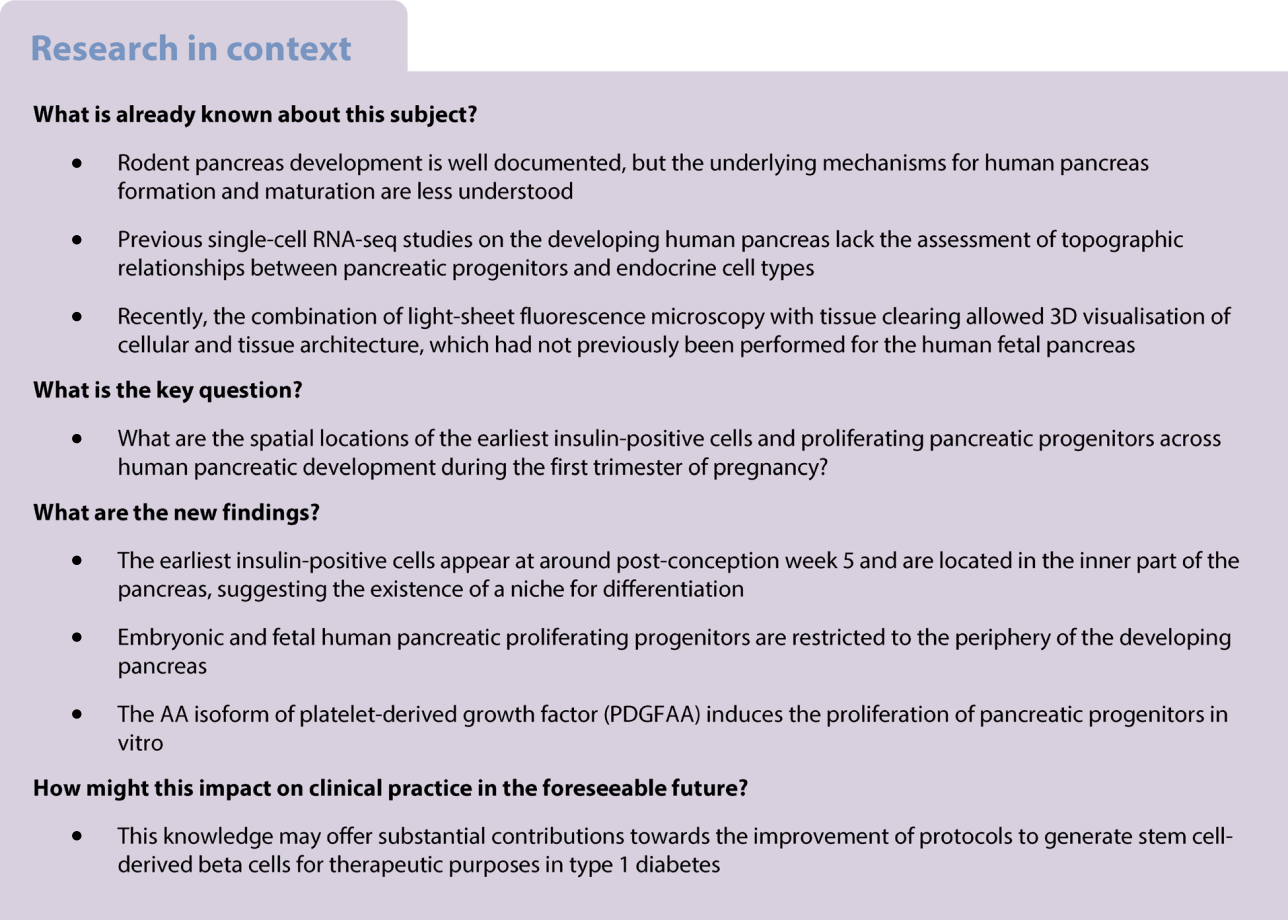


## Introduction

The pancreas is a vital organ involved in multiple crucial functions from glucose homeostasis to digestion [1]. It is a mixed gland composed of an exocrine and an endocrine fraction. The exocrine part contains acinar cells releasing enzymes like carboxypeptidase A1 (CPA1) and amylase through a ductal network draining into the duodenum. The endocrine fraction, clustered in the islets of Langerhans, accounts for 1% of the pancreatic mass. Pancreas dysfunction is associated with a large spectrum of disorders, including diabetes mellitus, pancreatitis and pancreatic cancer, which are major health burdens worldwide [2]. During embryonic development, the pancreas originates from the foregut endoderm and undergoes morphogenesis, characterised by extensive proliferation, migration and differentiation of distinct cell populations [3]. Among them, the endocrine cells, which produce hormones such as insulin (INS; beta cells), glucagon (alpha cells), and somatostatin (delta cells), are derived from a subset of pancreatic progenitor cells that undergo endocrine differentiation [1]. Understanding the regulation of this process is the subject of intense research, bearing significant implications for developmental biology and regenerative medicine [4, 5].

Rodent pancreas development has been studied in great detail during the past decades [6]. However, the developmental and cellular mechanisms underlying the formation and maturation of the human pancreas are far less understood. The development of the human pancreas begins during the fourth post-conceptional week (PCW), when dorsal and ventral pancreatic buds emerge from the endodermal cells of the developing gut tube [7]. These epithelial buds contain pancreatic progenitors that express key transcription factors such as pancreatic and duodenal homeobox 1 (PDX1), SRY-box transcription factor 9 (SOX9), NKX homeobox 1 (NKX6.1), forkhead box A2 (FOXA2) and pancreas-associated transcription factor A (PTF1A) [8]. They develop in an intricate fashion within the surrounding mesodermal-derived mesenchyme and undergo fusion, branching morphogenesis, differentiation of endocrine and exocrine cells, and establishment of functional connections with the surrounding organs [7]. Pancreatic progenitor cell differentiation into endocrine cells is tightly regulated by various transcription factors and signalling pathways [9]. In this context, the way the human pancreas develops represents a critical source of information [7].

Recently, the combination of light-sheet fluorescence microscopy (LSFM) and tissue clearing has enabled the three-dimensional (3D) visualisation of cellular and tissue architecture in unprecedented detail and resolution [10, 11]. Moreover, the integration of imaging data across different developmental stages has led to the creation of tissue atlases, providing comprehensive cellular maps [12].

So far, 2D descriptions have not captured the topographic relationship between pancreatic progenitors and endocrine cell types. In this study, we leveraged our expertise in LSFM and tissue clearing [13] to revisit the early stages of human pancreas development (PCW5–11), which retain their topographic integrity. We specifically aimed at investigating the spatiotemporal dynamics of INS^+^ cell differentiation and progenitor proliferation in the developing human pancreas using LSFM to build a human developing pancreas atlas. Our results reveal novel insights into the cellular mechanisms underlying pancreatic development and provide a foundation for future studies aimed at gaining further insights into human pancreas development.

## Methods

### Human and mouse embryonic and fetal pancreatic tissues

Human embryonic and fetal pancreatic tissues (PCW5–11) were obtained through the Inserm cross-cutting scientific programme Human Developmental Cell Atlas (HuDeCA) from surgical abortions in accordance with the French bioethics legislation and Inserm guidelines [14, 15]. Donors’ written consent was obtained, along with approval from Agence de Biomédecine, the competent authority in France. Fetal ages are displayed as PCW, staged based on morphometric correction [16]. All specimens were initially selected based on macroscopic morphological criteria, excluding samples with obvious malformations. In cases where visual determination of sex was not possible, PCR-based chromosome screening using DNA from biopsies was performed [13]. For labelling, a minimum of three samples per age were employed (electronic supplementary material [ESM] Table 1 and ESM Table 2), while for in vitro experiments we employed a minimum of five pancreases per condition.

Mouse fetal pancreases were obtained from pregnant C57BL/6J mice purchased from the Janvier Breeding Center (LeGenet, St Isle, France). Mice were killed by CO_2_ asphyxiation according to French Animal Care Committee guidelines and pancreases from embryos at embryonic day (E)10, E12 and E16 (at least six embryos per age) were dissected and fixed [17].

### Conventional 2D immunohistochemistry

Human embryonic and fetal pancreases were fixed in formalin, embedded in paraffin and sectioned (5 μm thick) as previously described [14]. The primary and secondary antibodies used are listed in ESM Table 3. EdU revelation was performed using the Click EdU Alexa 555 imaging kit (Thermo Fisher Scientific, Waltham, MA, USA). Nuclei were stained with Hoechst 33342 (0.3 mg/ml, Invitrogen, Waltham, MA, USA).

We manually counted the number of INS^+^ cells per cluster in all the 5 μm sections of three pancreases at PCW6 (180–240 sections/specimen). The percentage of PDX1^+^/KI67^+^ (at PCW7, 9 and 11) and INS^+^/KI67^+^ cells (at PCW9 and 11) were quantified on six sections per pancreas (3 pancreases/age). The spatial distribution of PDX1^+^/KI67^+^ progenitors was measured by computing the Ripley K function [*K*(*r*)] [18]. To do that, the PDX1^+^/KI67^+^ progenitors were mapped with *x*,*y* coordinates in 2D images at PCW7, 9 and 11 (minimum of *n*=20 images/developmental stage), using FIJI (version 2.14.0, Eliceirei/LOCI group, USA) [19]. Then, *K*(*r*) was computed for every image by implementing the R (version 4.1.2, R Foundation for Statistical Computing, Austria) library spatstat as previously described [20].

### 3D immunostaining

Embryonic and fetal samples were fixed by immersion in formalin at 4°C for 1 to 5 days depending on size. For tissue bleaching [10], fixed pancreases were dehydrated for 1 h at room temperature (RT) in increasing concentrations of methanol (50%, 80%, 100%) in 1X PBS. Then, the samples were incubated overnight at 4°C in a 6% hydrogen peroxide solution in 100% methanol, re-hydrated for 1hr at RT in decreasing concentrations of methanol (100%, 100%, 80%, 50%) and finally washed for 1 h in 1X PBS. Permeabilisation and blocking were performed under rotation at 70 rev/min at RT in PBSGT (1X PBS, 0.2% gelatin [Prolabo, Nemours, France], and 0.5% Triton X-100 [Sigma-Aldrich, Saint-Louis, MO, USA]) [13].

For whole-mount immunostaining, samples were incubated with the primary antibodies in a solution of PBSGT containing 0.1% saponin during 14 days at 37°C under rotation at 70 rev/min. The primary and secondary antibodies employed are listed in ESM Table 3. Then, tissues were incubated with the secondary antibodies in a solution of PBSGT (0.1% saponin [10 mg/ml]), during 4 days at 37°C under rotation at 70 rev/min. Next, samples were washed for 30 min in PBSGT at RT, embedded in 1.5% agarose (Roth, Goshen, IN, USA) prepared in TAE 1X (Invitrogen) [10, 11] and stored at 4°C until tissue clearing.

In order to achieve tissue transparency, the samples were cleared using a modification from the iDISCO+ protocol [21]. Fetal pancreases were dehydrated in growing concentrations of methanol (20%, 40%, 60%, 80%, 100%, 100%, 1 h each) at RT under rotation (14 rev/min), next incubated overnight in a solution of 2/3 dichloromethane (DCM) and 1/3 methanol, then for 30 min in 100% DCM, and finally transferred to dibenzyl ether (DBE) for further storage and imaging.

### 3D imaging and processing

Acquisition was performed with Miltenyi Biotec (Bergisch Gladbach, Germany) Ultramicroscope Blaze (sCMOS camera 5.5 MP) and Imspector Pro (version 7.3.2, LaVision Biotec, Germany) acquisition software. Laser light sheets are generated at excitation wavelengths of 488, 561, 640 and 785 nm. Lenses with 4× magnification (MI Plan 4× NA0.35) and 12× magnification (MI Plan NA0.53) objective were used. The *z*-step size is 3 µm. Images were generated as tiff files, converted to (.ims) by Imaris File Converter (version 9.8, Bitplane, UK), and the 3D images were reconstructed using the volume rendering function. To isolate a specific region of the tissue, the surface tool was used to manually segment. The 3D images, virtual slices and movies were generated using the snapshot and animation tools. To locate the pancreas in full intact PCW7 human embryo, the SOX9^+^ pancreatic area was delimited in consecutive slices.

Human and mouse pancreatic lengths and diameters were measured with the distance tool in virtual slices. The measurements of the SOX9^+^ and INS^+^ cluster volume and density were performed following the automatic segmentation tool. The distribution of human INS^+^ clusters was measured in three virtual slices from three different pancreases. First, the pancreatic epithelium was manually outlined and the centroid was determined for each slice. Then, the distance from every INS^+^ cluster was measured to the centroid [19].

### Tissue culture

Whole human embryonic and fetal pancreases at PCW6–10 were cultured for a maximum of 7 days at an air-complete medium interface in Petri dishes on 0.45 μm filters (Millipore) [22]. Explants were cultured in G10 medium consisting of RPMI 1640 + glutaMax, 10% FBS, 1% penicillin/streptomycin, 1% HEPES and 1% non-essential amino-acids (61870036, ThermoFisher Scientific). Medium was replaced every 2 days. For EdU incorporation assays, pancreases were treated with 5 µmol/l EdU for 4 h, the reaction was stopped by washing out the tissue with G10 medium and immediate fixation. For experiments with the platelet-derived growth factor (PDGF) AA isoform (PDGFAA), explants were cultured in DMEM/F12 medium (31331, ThermoFisher Scientific) containing ALK5 inhibitor (ALK5i; 10 mmol/l, 221234, Chem Cruz, Dallas, TX, USA), noggin (500 mg/ml, 120–10C, Peprotech, Waltham, MA, USA) and nicotinamide (2.5 mol/l, 481907, Sigma). Explants were treated with PDGFAA (200 ng/ml, PHG0035, Sigma) for 7 days, replacing the medium every 2 days.

## Results

### Human embryonic and fetal pancreases display isometric growth from 5 to 11 PCW

**Fig. 1:**
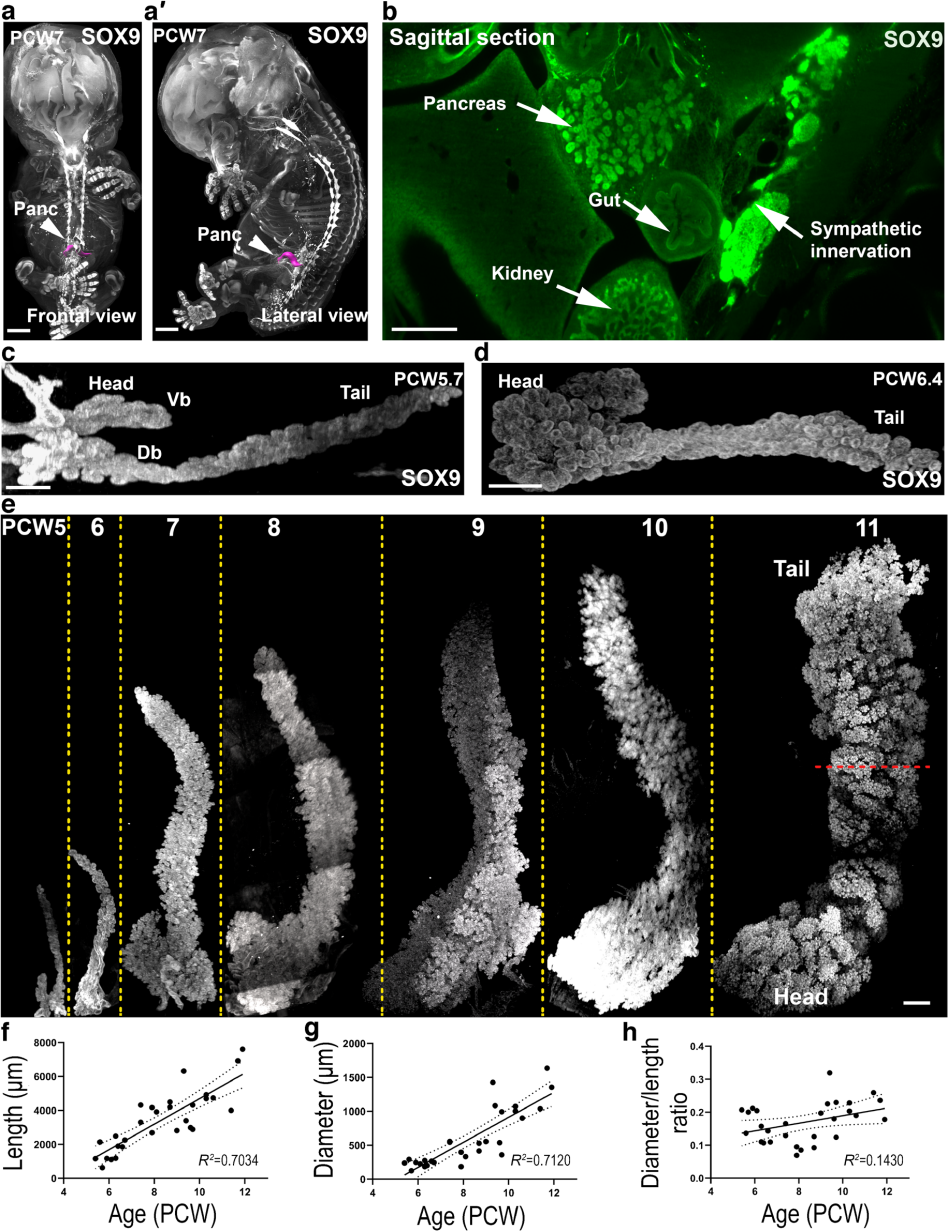
Human embryonic and fetal pancreases display isometric growth from PCW5 to 11. LSFM images of embryonic and fetal pancreases (**a**–**e**) immunostained for SOX9 or SOX9 and TH (**a**, **a′**, **b**). (**a**, **a′**) Frontal and lateral 3D views of a PCW7 human embryo revealing the anatomical location of the human pancreas (magenta). SOX9 is expressed in the cartilage and various organs including the pancreas. (**b**) Sagittal *z*-stack section of the PCW7 human embryo from Fig. 1a locating the pancreas and adjacent tissues. (**c**, **d**) At PCW5.7, SOX9 staining labels both ventral and dorsal buds, which have fused at PCW6.4. (**e**) Tridimensional SOX9 immunostaining (white) in whole-mount pancreases dissected from human embryos and fetuses between PCW5 and 11. Dashed red line depicts the diameter of the organ. (**f**–**h**) Determination of the length (**f**), the diameter (**g**), and the diameter to length ratio (**h**) of the pancreases from PCW5–11. Scale bars: 2 mm (**a**, **a′**), 500 µm (**b**, **e**), 200 µm (**c**, **d**). Db, dorsal bud; Panc, pancreas; TH, tyrosine hydroxylase; Vb, ventral bud


Significant progress in conventional 2D immunostaining has provided highly valuable information about human pancreas development [8]. However, there is limited work providing 3D analyses. Here, we report for the first time the use of iDISCO+ [21] tissue clearing and LSFM to track the morphological development of the human embryonic and fetal pancreas. Conventional immunostainings indicated that pancreatic progenitors are immunoreactive for the transcription factors SOX9, PDX1 and NKX6.1 [7]. We performed triple staining that demonstrated similar pancreatic expression patterns for all three markers in both conventional immunostaining (ESM Fig. 1a) and 3D imaging (ESM Fig. 1b and ESM Video 1). Therefore, during this developmental period PCW5–11, these three markers (SOX9, PDX1 and NKX6.1) can be interchangeably used to identify progenitors. SOX9 staining, in an intact transparent PCW7 human embryo, allowed us to precisely visualise the pancreas in the upper left abdomen, highlighted in magenta (Fig. 1a,b and ESM Video 2). We next immunolabelled PCW5–11 pancreas for SOX9, followed by clearing and LSFM. In the mouse, the pancreas develops from two independent buds that will fuse later on [23]. However, the timing of this process was poorly documented in humans. At PCW5, both ventral and dorsal buds were detected (Fig. 1c, and ESM Video 3), whereas at PCW6 they had fused and formed a single pancreatic primordium (Fig. 1d).

**ESM Video 1, related to ESM Fig. 1** Expression pattern of SOX9, PDX1 and NKX6.1 in the human fetal pancreatic epithelium. Staining of SOX9 (cyan), PDX1 (magenta) and NKX6.1 (yellow) in the human pancreatic epithelium at PCW8. Supplementary file2 (MP4 88758 KB)**ESM Video 2, related to Fig. 1** Anatomical location of the pancreas in the human embryo. Human embryo at PCW7 stained with SOX9 and TH in white. The pancreas is highlighted in magenta. Supplementary file3 (MP4 53677 KB)**ESM Video 3, related to Fig. 1** Detection of ventral and dorsal buds with light-sheet fluorescence microscopy in human embryonic pancreas at PCW5.7. SOX9 in white. Supplementary file4 (MP4 53982 KB)

3D imaging in our PCW5–11 series revealed an obvious increase in pancreatic size (Fig. 1e). To quantify human pancreas growth, we measured their lengths along the longitudinal axis and diameters across the dorso-ventral axis. Both length and diameter increased across development from PCW5 to PCW11 (*n*=29 pancreases) (Fig. 1f,g). The growth in both length and diameter showed a sixfold increase from PCW5 to PCW11, revealing a positive linear correlation between pancreatic size and age. The quantification of the diameter to length ratio showed that it remained stable (Fig. 1h), indicating that pancreas growth is homothetic during the first trimester of gestation.

### INS^+^ cells arise around PCW5 and are located in the centre of the human developing pancreas

**Fig. 2:**
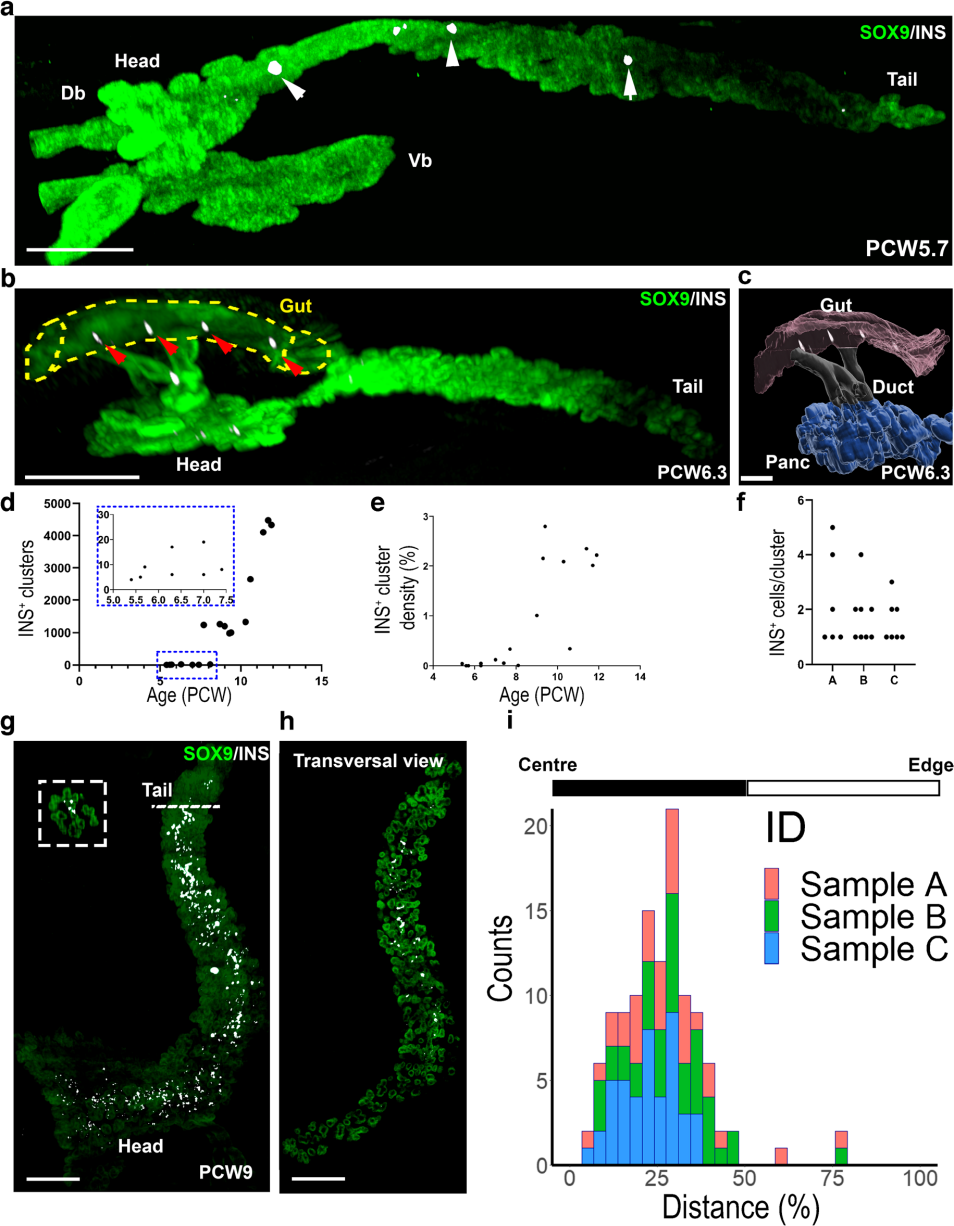
Spatial and time determination of INS^+^ cell location in the pancreatic primordium. LSFM images of embryonic and fetal pancreases (**a**, **b**, **g**, **h**) immunostained for SOX9 (green) and INS (white). (**a**) At PCW5.7, a few clusters of insulin-immunoreactive (INS^+^) cells (see white arrows) are found at the centre of the dorsal bud. (**b**) Representative image of extra-pancreatic INS^+^ cells that were detected in a gut region adjacent to the head of the pancreas (see red arrows) (PCW 6.3) (*n*=4 pancreases). (**c**) Segmentation of the pancreas, the gut tube and ducts from Fig. 2b, showing the gut region next to the pancreas containing INS^+^ clusters. (**d**) Determination of the number of INS^+^ clusters from PCW5–11. (**e**) Determination of INS^+^ cluster density (volume of INS^+^ cluster /volume SOX9^+^ epithelium) from PCW5–11. (**f**) Quantification of the number of INS^+^ cells per cluster at PCW6, full sectioned and stained to include all the INS^+^ clusters (*n*=3) in three different samples (named A, B, and C). (**g**) At PCW9, INS^+^ clusters are aligned to the centre of the pancreas as shown in 3D. The dashed line represents a sagittal section. (**h**) Transversal view of Fig. 2g. (**i**) Histogram showing the distribution of the INS^+^ clusters across the pancreatic epithelium in 2D sections from three PCW8 specimens (samples A, B and C). The *x*-axis represents the distance of the INS^+^ clusters to the centre of the pancreas. Scale bars: 250 µm (**a**), 300 µm (**b**), 50 µm (**c**), 200µm (**g**, **h**). Db, dorsal bud; Panc, pancreas; Vb, ventral bud


3D imaging allows the precise spatial location of specific pancreatic cell populations. We next asked when and where the first INS^+^ cells appear. Previous reports on human fetal pancreases based on 2D immunohistochemistry detected the earliest INS^+^ cells around PCW7 [8]. Here, we were able to detect the presence of INS^+^ clusters as early as PCW5.7 (Fig. 2a). At this stage, they were restricted to the dorsal bud and absent from the ventral bud (Fig. 2a, and ESM Video 4), indicating that, as in the mouse [24], INS^+^ cells first appear in the dorsal pancreas in humans. Recent studies by conventional 2D immunostaining revealed the existence of INS^+^ cells in the human fetal gut between PCW10 and PCW19 [25]. We could detect extra-pancreatic INS^+^ clusters far earlier between PCW6.3 and PCW10.6, in the fetal gut next to the pancreas (Fig. 2b,c, and ESM Video 5).

**ESM Video 4, related to Fig. 2** Temporal location of the first INS+ cells in the human embryonic pancreas. Pancreas at PCW5.7 stained with SOX9 in green and INS in white. Supplementary file5 (MP4 83252 KB)**ESM Video 5, related to Fig. 2** Detection of extra-pancreatic INS+ cells in the human embryonic gut with light-sheet fluorescence microscopy. Pancreas at PCW6.3 stained with SOX9 in green and INS in white. Supplementary file6 (MP4 25740 KB)

We next quantified the number of pancreatic INS^+^ clusters on 19 samples between PCW5.4 and PCW11. We observed few INS^+^ clusters from PCW5.4 to 7.5 (9.25±5.65, mean ± SD, *n*=8), with a sharp increase at later stages (4307±152.34, mean ± SD, *n*=3 at PCW11) (Fig. 2d,e). We also manually quantified, using conventional immunostaining, the number of INS^+^ cells per cluster in paraffin sections from three pancreases (180–240 sections per specimen) at PCW6. We found that the first INS^+^ clusters contain between 1 and 5 INS^+^ cells (Fig. 2f).

We next analysed the location of these INS^+^ clusters and observed their preferential location in the centre of the pancreas, being almost absent from the periphery. INS^+^ clusters were aligned along the longitudinal axis of the pancreas (Fig. 2g,h and ESM Video 6). We mapped in 3D the distribution of INS^+^ clusters to the centroid of the SOX9^+^ pancreatic epithelium in *z*-slices from 3D scans from three pancreases at PCW9. In all three pancreases, the vast majority of the INS^+^ clusters were located closer to the centre rather than to the periphery (Fig. 2i).


**ESM Video 6, related to Fig. 2** Spatial location of INS+ clusters in the human fetal pancreas. Pancreas at PCW8 stained with SOX9 in green and INS in white. Supplementary file7 (MP4 106204 KB)

Our data thus strongly suggest the existence of a niche in the centre of the pancreas promoting the differentiation of progenitors towards an INS^+^ cell fate. Given that vascularisation has been suggested as an important player in beta cell development in mice [26], we mapped vascular markers with regards to INS^+^ cells in the human embryonic/fetal pancreas. The general marker for mature vascular endothelial cells CD34 was expressed in a uniform network of vessels intermingled with SOX9^+^ pancreatic progenitors, without obvious differential distribution from centre to peripheral domains (ESM Fig. 2a). Noticeably, the whole extent of the developing pancreas was devoid of the more specific arterial marker smooth muscle α-actin (SMA) at PCW9 (ESM Fig. 2b).

Finally, we compared pancreatic development between humans and mice by LSFM (ESM Fig. 3a, c). As expected, the number of INS^+^ clusters increased in mice from E10 to E16 (ESM Fig. 3d and ESM Table 4). Likewise, pancreatic length and diameter increased in mouse embryos between E12 and E16 (ESM Fig. 3e, f). However, the diameter/length ratio increased across development (ESM Fig. 3g), which is different from what we observed in human pancreatic samples from PCW5–11.

### Proliferating pancreatic progenitors are restricted to the periphery of the epithelium

**Fig. 3:**
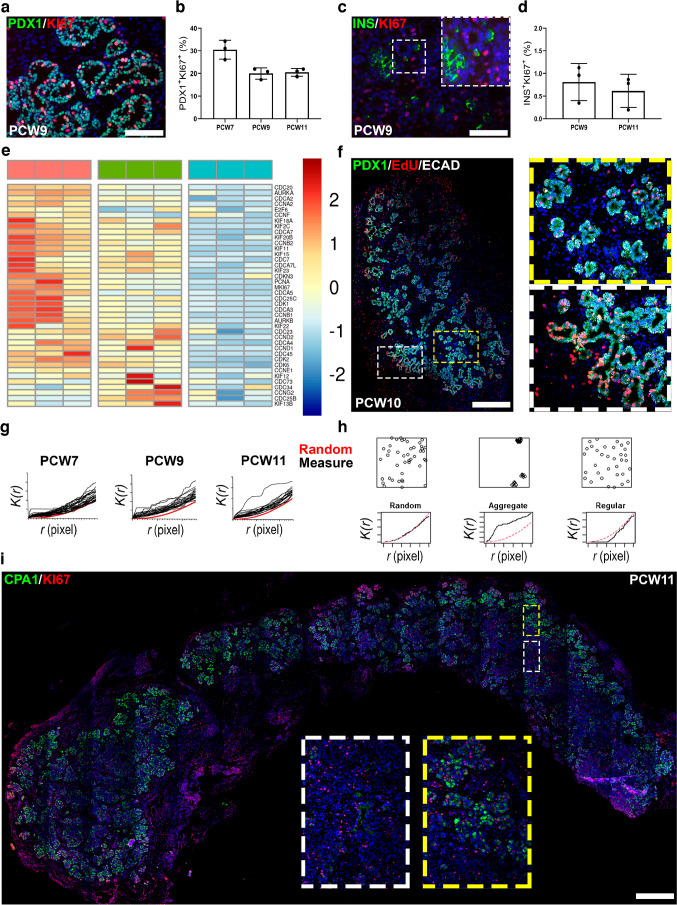
Proliferation of human embryonic and fetal pancreatic cells. (**a**, **b**) Representative staining for PDX1 (green) and KI67 (red) on sections of human fetal pancreas at PCW9 and quantification of the frequency of PDX1^+^/Ki67^+^ at PCW7, 9 and 11 (*n*=3). (**c**, **d**) Representative staining for INS (green) and KI67 (red) on sections of human fetal pancreas at PCW9 and quantification of the frequency of INS^+^/Ki67^+^ at PCW9 and 11. Inset reveals a single INS^+^KI67^+^ cell. (**e**) Heatmap showing the expression of cell cycle-related genes in three successive populations of pancreatic progenitors: ECAD^+^CD142^+^ (pink); ECAD^+^CD142^−^ (green); ECAD^low^CD142^−^SUSD2^+^ (blue). The heatmap was generated by the ‘heatmap2’ function from gplots R package (https://cran.r-project.org/web/packages/gplots/) on standardised log_2_ expression values from Ramond et al 2018 [15], with Pearson correlation as the distance function. (**f**) Proliferating pancreatic progenitors (PDX1^+^/EdU^+^) across the pancreatic epithelium. PDX1 in green, EdU in red and ECAD in white (*n*=6). (**g**) Computation of the Ripley K function [*K*(*r*), with *r* being the distance in pixels from one cell to the closest one] to determine the distribution pattern of PDX1^+^KI67^+^ progenitors in 2D images at PCW7, 9 and 11. Red lines represent the random distribution while each black line represents the quantification of *K*(*r*) for PDX1^+^KI67^+^ progenitors in a single 2D image. (**h**) Expected output of *K*(*r*) computed for three different types of spatial distribution: random, aggregate and regular. (**i**) Determination of the cell identity of peripheral pancreatic cells. CPA1 in green and KI67 in red in a human fetal pancreas at PCW11. Scale bars: 100 µm (**a**, **c**), 500 µm (**f**), 1 mm (**i**)

Next, we sought to correlate human pancreatic differentiation dynamics with proliferation kinetics across the first trimester. KI67/PDX1 double staining indicated that 20–30% (*n*=3) of pancreatic progenitors (PDX1^+^) are in a proliferative state during PCW7–11 (Fig. 3a,b) while differentiated INS^+^ cells proliferate at a far lower rate at a ratio around 1% at PCW 9–11 (*n*=3) (Fig. 3c,d).

By selecting cell surface markers and using FACS, we had previously subdivided human fetal pancreatic progenitors into a number of populations with epithelial cadherin (ECAD)^+^ CD142^+^ sushi domain containing 2 (SUSD2)^−^ giving rise to ECAD^+^CD142^−^SUSD2^–^ and finally to ECAD^low^CD142^−^SUSD2^+^ [15]. Here, we first reanalysed our transcriptomic datasets [15], asking whether proliferation markers change from one progenitor population to the other. By using our previously validated list of pancreatic proliferation markers [27], we observed a downregulation from population ECAD^+^CD142^+^SUSD2^−^ to ECAD^low^CD142^−^SUSD2^+^, including canonical proliferation genes such as *KI67* (also known as *MKI67*), *PCNA* and *CDK1* (Fig. 3e). This indicates that progenitors proliferate at different rates in the human embryonic/fetal pancreas and proliferation decreases along with endocrine differentiation. The next question concerned the spatial location of proliferating progenitors. We took advantage from recurrent access to fresh pancreases and used EdU, a thymidine analogue, to label cells entering S-phase. Proliferating pancreatic progenitors (PDX1^+^EdU^+^) were found to be highly enriched at the periphery of the pancreas, while their frequency in the centre was extremely low (Fig. 3f). The distribution pattern of pancreatic proliferating progenitors was mathematically analysed by computing the Ripley K function [*K*(*r*)] [18] in 2D images at PCW7, 9 and 11. It showed that PDX1^+^KI67^+^ progenitors are clustered together in aggregates, rather than randomly distributed (Fig. 3g,h). We finally determined whether proliferating PDX1^+^KI67^+^ cells might represent acinar cells that express CPA1. We observed that the human fetal pancreas is enriched in CPA1^+^ cells in the periphery, rather than in the centre (Fig. 3i), further supporting that the inner fetal pancreas is an endocrine niche. We also found that the majority of these CPA1^+^ cells lacked KI67 expression. Hence, the fact that most of the proliferating peripheral cells are not CPA1^+^ sustains the idea that these cells are progenitors rather than differentiated acinar cells. Overall, our data further support the existence of a niche of proliferation for pancreatic progenitors in the periphery of the human embryonic and fetal pancreas.

### PDGFAA induces the proliferation of human pancreatic progenitors in vitro

**Fig. 4:**
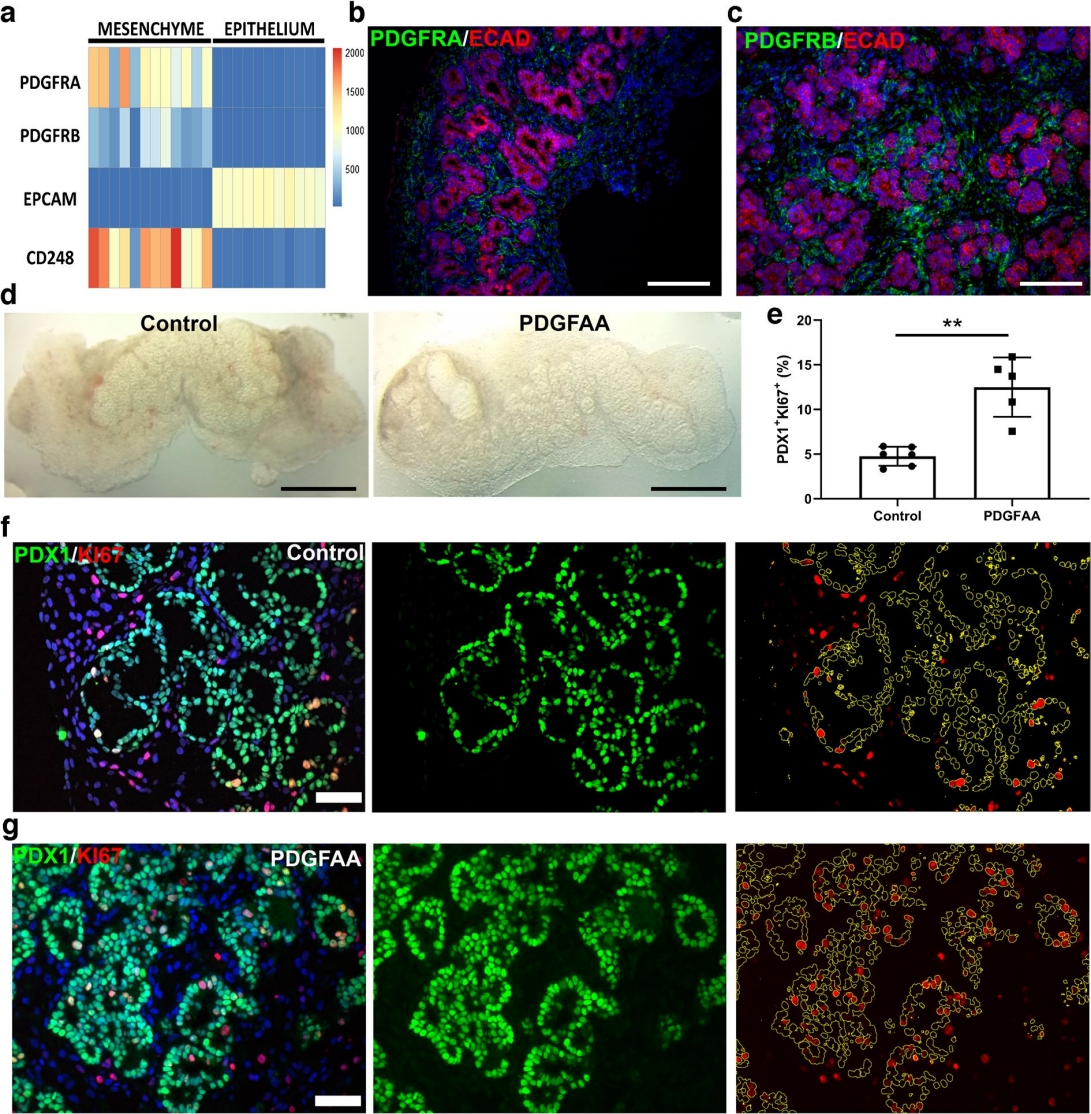
Effect of PDGFAA in explants of human embryonic and fetal pancreases. (**a**) Heatmap showing the expression of PDGFRA and PDGFRB in the mesenchymal and epithelial fraction of human fetal pancreases. Epithelial (epithelial cellular adhesion molecule [EPCAM]) and mesenchymal (CD248) markers are used as positive controls for each population. The heatmap was generated as described in the legend for Fig. 3e with expression values from Ramond et al 2017 [14]. (**b**, **c**) Expression pattern of PDGFRA and PDGFRB (green) in a human fetal pancreas at PCW10, ECAD is in red. (**d**) Macroscopic integrity of the tissue at day 7 of culture in control or treated samples. (**e**) Determination of the proliferation ratio for pancreatic progenitors at day 7 cultured in DMEM/F12 supplemented medium treated or not with PDFGAA (*n*=5–6). Mean ± SD. (**f**, **g**) Representative images of control and treated (PDGFAA) samples at day 7 cultured in DMEM/F12 supplemented medium stained with PDX1 (green) and KI67 (red). Scale bars: 150 µm (**b**, **c**, **f**, **g**), 1 mm (**d**)

We finally searched for signalling pathways that induce the proliferation of human pancreatic progenitors. For this purpose, we cultured human fetal pancreases over a floating filter disk in a gas–liquid interface for either 3 or 7 days. Under such conditions, pancreatic progenitors were maintained up to 7 days keeping a similar proliferation ratio (16.94±7.72% and 14.54±0.78% of PDX1^+^ cells at day 3 and 7, respectively, mean ± SD, *n*=5–6 per group) than in vivo (19.98±2.42%, mean ± SD, *n*=3 at PCW9) (ESM Fig. 4a). Furthermore, the progenitors kept their multipotent phenotype during the full culture period, as assessed by the expression of PDX1 and NKX6.1 (ESM Fig. 4b). Gain- and loss-of-function experiments using either fibroblast growth factor (FGF)10 [28] or the mitogen-activated protein kinase kinase 1 (MEK1) inhibitor PD98059 [29], respectively, did not alter the PDX1^+^KI67^+^ ratio (data not shown). We next switched to a culture medium that contains ALK5i, noggin and nicotinamide that showed a reduction in the PDX1^+^/KI67^+^ ratio to around 5% to be used to search for factors that enhance progenitor proliferation. Our previous transcriptomic data [14] indicated that PDGF receptor α (PDGFRA) and PDGF receptor β (PDGFRB) are specifically expressed by human fetal pancreatic mesenchyme but not in pancreatic epithelial cells (Fig. 4a) and we further confirmed such data by immunostaining (Fig. 4b,c). We thus treated pancreatic explants with PDGFAA for 7 days. The macroscopic architecture of the explants remained unaffected (Fig. 4d). We observed a threefold increase in the number of proliferating progenitors (PDX1^+^KI67^+^) (Fig. 4e). We also observed changes in the microscopic tissue architecture following PDGFAA treatment. While the lumen size increased in control explants during the culture period, upon PDGFAA treatment, their size became more similar to that observed in vivo (Fig. 4f,g). Overall, through the establishment of a culture model of human fetal pancreases, we suggested that PDGF acts indirectly through mesenchymal cells to induce progenitor cell proliferation.

## Discussion

There is an urgent need to improve the development of stem cell-derived human beta cells, to build novel therapies for the treatment of type 1 diabetes [30]. Most of the current protocols attempt to recapitulate beta cell neogenesis processes [31, 32]. However, the vast majority of our knowledge on beta cell differentiation comes from studies on mouse pancreas development [3, 33] and a detailed timeline and topographic description of human embryonic and fetal pancreatic cell proliferation and differentiation remained to be established. Here, through the Inserm-funded HuDeCA consortium (https://hudeca.genouest.org/), we could access a large collection of fresh pancreases between PCW5 and PCW11.

The majority of previous histological data on the developing mouse and human pancreas has been generated using conventional 2D sections [7, 8, 34]. Recently, a 3D description of the developing mouse pancreas was published [35]. It provided a comprehensive view of the cellular composition and topological relationships of embryonic pancreatic cells. Here, we employed a similar pipeline to generate the first 3D atlas of the developing human pancreas by in toto staining, tissue clearing and LSFM imaging. We focused on the first trimester, when first endocrine cells are generated [7]. An interesting observation is our detection of two pancreatic buds, ventral and dorsal, prior to their fusion. We also detected the first INS^+^ cells in the dorsal pancreas, similar to what has been described in mouse and zebrafish embryonic pancreases [24, 36]. Here we could detect INS^+^ cells at around PCW5, 2 weeks earlier than previously described [8]. We also confirm the existence of extra-pancreatic INS^+^ cells in the gut region adjacent to the pancreas [25].

Our data show that the earliest INS^+^ cells are either isolated or form very small clusters (of about 4–5 cells), when in adults, islets contain hundreds of INS^+^ cells. Using KI67, we found that human embryonic and fetal INS^+^ cells exhibit a low proliferation rate. This suggests that islets do not form by proliferation in situ of preexisting INS^+^ cells, consistent with our previous data [37]. Additionally, similar observations from mouse chimeras further support the polyclonal origin of pancreatic islets [38, 39]. We thus postulate that in humans, mature islets form by the aggregation of preexisting INS^+^ cells. Mouse data suggest that endocrine cell differentiation is followed by epithelial–mesenchymal transition, migration and aggregation [40]. However, in an alternative hypothesis, INS^+^ cells could differentiate from endocrine progenitors located within a single epithelial cord while maintaining contact with this structure, forming a peninsula and then an islet [41].

We raised questions regarding the earliest INS^+^ cells and their location. INS^+^ cells were preferentially located in the centre of the pancreas where they were tightly aligned, while almost absent in the periphery, suggesting the existence of a highly specific differentiation niche for endocrinogenesis in the inner part of the pancreas. We can speculate that endocrine progenitors expressing the transcription factor neurogenin3 (NGN3) are also located in the centre of the developing human pancreas. However, we could not validate experimentally that point as we were unable to find a suitable NGN3 antibody compatible with our 3D staining approach. Signals controlling this specific spatial pattern of differentiation remain unknown. Although vascularisation has been shown to play major roles in mouse beta cell development [26, 42, 43], we did not observe a correlation between the location of INS^+^ cells and the vascular tree. Whether the specific location of the first human INS^+^ cells is influenced by nerves, an important player in beta cell physiology [44], remains to be explored. We are also testing the hypothesis that the mesenchyme located in the inner and peripheral regions of the pancreas are different.

Our KI67 data indicate that the proliferation rate of INS^+^ cells is extremely limited during the second part of the first trimester of gestation, about 20 times lower than pancreatic progenitors. This demonstrates that the amplification pool for endocrinogenesis is at the level of pancreatic progenitors and not INS^+^ cells. This is in agreement with data obtained in mouse embryos where fetal pancreatic progenitors proliferate far more efficiently than beta cells and their number is increased by growth factors such as FGF7 and FGF10 [22, 29]. We observed that, in contrast with INS^+^ cells, progenitor proliferation is restricted to the periphery of the pancreas. These findings suggest that endocrine cell differentiation and progenitor proliferation are differentially regulated processes during human pancreatic development.

Here, we developed a culture system to study human progenitor cell proliferation, using a strategy previously used in rat embryos [22]. Accordingly, we found that in human embryonic pancreas cultures progenitors proliferate for at least 7 days at a similar ratio to in vivo. By using this model, we demonstrate that PDGFAA, a molecule produced in the fetal pancreas by epithelial [45] and vascular smooth muscle [46] cells, enhances the proliferation of pancreatic progenitors. Recently, *PDGFRA* mRNA was found to be enriched in the pancreatic mesenchyme [45, 46], in agreement with our transcriptomic data [14] and with single cell transcriptomic data derived from the DESCARTES Atlas (https://descartes.brotmanbaty.org/, last accessed 7 February 2024). We confirmed here that the expression of PDGFRA and PDGFRB is restricted to the pancreatic mesenchyme, suggesting that PDGF exerts an indirect influence on progenitors by affecting mesenchymal cells.

Altogether, we present here the first attempt to build a 3D atlas of the developing human pancreas. Overall, this resource sheds light on the genesis and location of the first INS^+^ cells, as well as pancreatic proliferating progenitors. Furthermore, we also describe the mitogenic effect of PDGFAA in the pancreatic epithelium. Our 3D imaging pipeline will allow researchers to explore the developing anatomy of the pancreas in a spatial context, and to visualise the relationships between different structures and cell types. Thus, this 3D atlas should be further expanded by mapping other endocrine cells such as glucagon- and somatostatin-expressing cells. This information holds great potential for enhancing our understanding of the underlying mechanisms driving embryonic and fetal pancreatic development, and potentially this knowledge could further improve the generation of stem cell-derived beta cells.

### Supplementary Information

Below is the link to the electronic supplementary material.

Supplementary file7 (PDF 1.15 MB)

**ESM Video 1, related to ESM Fig. 1** Expression pattern of SOX9, PDX1 and NKX6.1 in the human fetal pancreatic epithelium. Staining of SOX9 (cyan), PDX1 (magenta) and NKX6.1 (yellow) in the human pancreatic epithelium at PCW8. Supplementary file2 (MP4 88758 KB)

**ESM Video 2, related to Fig. 1** Anatomical location of the pancreas in the human embryo. Human embryo at PCW7 stained with SOX9 and TH in white. The pancreas is highlighted in magenta. Supplementary file3 (MP4 53677 KB)

**ESM Video 3, related to Fig. 1** Detection of ventral and dorsal buds with light-sheet fluorescence microscopy in human embryonic pancreas at PCW5.7. SOX9 in white. Supplementary file4 (MP4 53982 KB)

**ESM Video 4, related to Fig. 2** Temporal location of the first INS+ cells in the human embryonic pancreas. Pancreas at PCW5.7 stained with SOX9 in green and INS in white. Supplementary file5 (MP4 83252 KB)

**ESM Video 5, related to Fig. 2** Detection of extra-pancreatic INS+ cells in the human embryonic gut with light-sheet fluorescence microscopy. Pancreas at PCW6.3 stained with SOX9 in green and INS in white. Supplementary file6 (MP4 25740 KB)

**ESM Video 6, related to Fig. 2** Spatial location of INS+ clusters in the human fetal pancreas. Pancreas at PCW8 stained with SOX9 in green and INS in white. Supplementary file7 (MP4 106204 KB)

## Data Availability

The most relevant data generated or analysed during this study are included in this manuscript and the ESM. Further datasets generated during the current study are available from the corresponding author upon request.

## Abbreviations

ALK5i: ALK5 inhibitor
CPA1: Carboxypeptidase A1
ECAD: Epithelial cadherin
FGF: Fibroblast growth factor
HuDeCA: Human Developmental Cell Atlas
INS: Insulin
LSFM: Light-sheet fluorescence microscopy
NKX6.1: NKX homeobox 1
PCW: Post-conceptional week(s)
PDGF: Platelet-derived growth factor
PDGFAA: PDGF AA isoform
PDGFRA: PDGF receptor α
PDGFRB: PDGF receptor β
PDX1: Pancreatic and duodenal homeobox 1
RT: Room temperature
SOX9: SRY-box transcription factor 9
SUSD2: Sushi domain containing 2
