# Supplementary material for: A 3D atlas of the human developing pancreas to explore progenitor proliferation and differentiation
Source: Diabetologia. 2024 Apr 17;67(6):1066–78. doi: 10.1007/s00125-024-06143-2 (PMC11058870; doi:10.1007/s00125-024-06143-2)
Supplement: Supplementary file 7 — Supplementary file7 (PDF 1.15 MB) [file 125_2024_6143_MOESM7_ESM.pdf]

Tables

ESM Table 1. Human embryonic and fetal pancreases grouped by age employed in 3D immunostaining.

Age range (PCW)	Specimen number	Age (PCW)
**5–5.9**	EH3915	5.4
	EH3901	5.6
	EH3912	5.7
	EH4181	5.9
**6–6.9**	EH3852	6.1
	EH3464	6.3
	EH3785	6.3
	EH3333	6.4
	EH3838	6.6
	EH3374	6.7
**7–7.9**	EH3363	7.4
	EH3674	7.4
	EH3622	7.9
	EH3378	7.9
**8–8.9**	EH3575	8.1
	EH3058	8.7
	EH3079	8.7
**9–9.9**	EH4001	9.0
	EH3984	9.3
	EH3094	9.4
	EH3127	9.6
	EH3104	9.7
	EH3460	9.7
**10–10.9**	EH3839	10.3
	EH4155	10.3
	EH3950	10.6
**11–11.9**	EH3366	11.4
	EH3084	11.7
	EH3835	11.9

A total of 29 human embryonic and fetal tissues from PCW5–11 were used for the experiments of 3D immunostaining, with a minimum of three samples per age.

ESM Table 2. Human embryonic and fetal pancreases represented in each figure and panel.

Figure	Panel	Specimen Number	Age (PCW)	Sex	Staining
1	A-B	Ac713	7.1	Male	SOX9/TH
1	C	EH3912	5.7	Male	SOX9
1	D	EH3333	6.4	Male	SOX9
1	E	EH3912 (PCW5)	5.7	Male	SOX9
1	E	EH3374 (PCW6)	6.7	Male	SOX9
1	E	EH3674 (PCW7)	7.4	Male	SOX9
1	E	EH3079 (PCW8)	8.7	Male	SOX9
1	E	EH3984 (PCW9)	9.3	Female	SOX9
1	E	EH3950 (PCW10)	10.6	Male	SOX9
1	E	EH3084 (PCW11)	11.7	Male	SOX9
2	A	EH3912	5.7	Male	SOX9/INS
2	B-C	EH3364	6.3	Female	SOX9/INS
2	G-H	EH4201	9.0	Male	SOX9/INS
3	A	EH3522	9.4	Female	PDX1/KI67
3	C	EH3522	9.4	Female	PDX1/INS
3	F	EH4150	10.0	Female	PDX1/ECAD/EdU
3	I	EH4015	11.4	Female	CPA1/KI67
4	B-C	EH3630	10.1	Female	PDGFRA/PDGFRB/ECAD
4	F	EH4342	8.7	Male	PDX1/KI67
4	G	EH4360	9.3	Female	PDX1/KI67
ESM Fig. 1	A	EH4091	8.1	Male	SOX9/PDX1/NKX6.1
ESM Fig. 1	B	EH3524	8.3	Male	SOX9/PDX1/NKX6.1
ESM Fig. 2	A	EH3155	8.7	Male	SOX9/INS/CD34
ESM Fig. 2	B	EH3877	9.3	Male	SOX/INS/SMA
ESM Fig. 3	A	EH4255	8.7	Female	PDX1/KI67
ESM Fig. 3	B	EH4300	7.0	Male	PDX1/NKX6.1
Movie 1		EH4089	8.3	Female	SOX9/PDX1/NKX6.1
Movie 2		AC713	7.1	Male	SOX9/TH
Movie 3		EH3912	5.7	Male	SOX9
Movie 4		EH3912	5.7	Male	SOX/INS
Movie 5		EH3364	6.3	Female	SOX/INS
Movie 6		EH3674	7.4	Male	SOX/INS

Each specimen appearing in the figures is labelled and records the sex information.

ESM Table 3. Antibody list.

Antibody	Reference	Dilution	Source
mouse anti-insulin	I2018	1:1,000	Sigma-Aldrich
goat anti-SOX9	AF3075	1:1,000	R&D Systems
mouse anti-ECAD	610182	1:200	BD Biosciences
mouse anti-NKX6.1	F55A12	1:1,000	DSHB
mouse anti-KI67	550609	1:100	BD Biosciences
rabbit anti-PDX1	[47]	1:1,000	Homemade
rabbit anti-PDGFRA	ab124392	1:500	Abcam
anti-PDGFRB	ab32750	1:500	Abcam
goat anti-CPA1	AF2856	1:400	R&D Systems
goat anti-SOX9	AF3075	1:5,000	R&D Systems
guinea pig anti-insulin	A0564	1:5,000	Dako
rabbit anti-SMA	ab5694	1:1,000	Abcam
mouse anti-CD34	ab8536	1:5,000	Abcam
rabbit anti-TH	ab137869	1:1,000	Abcam
anti-rabbit Alexa Fluor 488	A11034	1:400	Life Technologies
anti-rabbit Alexa Fluor 555	ab150062	1:400	Abcam
anti-mouse Alexa Fluor 488	ab150105	1:400	Abcam
anti-mouse Alexa Fluor 594	115-585-003	1:400	Jackson ImmunoResearch
anti-mouse Alexa Fluor 647	ab150111	1:400	Abcam
anti-goat Alexa Fluor 488	ab150129	1:400	Abcam
anti-guinea pig Alexa Fluor 488	1:500	A-11073	Thermo Fisher Scientific
donkey anti-mouse Alexa Fluor 555	ab150110	1:500	Abcam
donkey anti-rabbit 647	ab150063	1:500	Abcam
donkey anti-rabbit 790	ab186693	1:250	Abcam
donkey anti-goat Alexa Fluor 790	705-655-147	1:250	Jackson ImmunoResearch

ESM Table 4. Comparison of human and murine pancreatic development.

**DIFFERENTIATION**	**Murine**	**E10**	**E12**	**E16**
		-	+	+++
	**Human**	**PCW5**	**PCW8**	**PCW11**
		-	+	+++

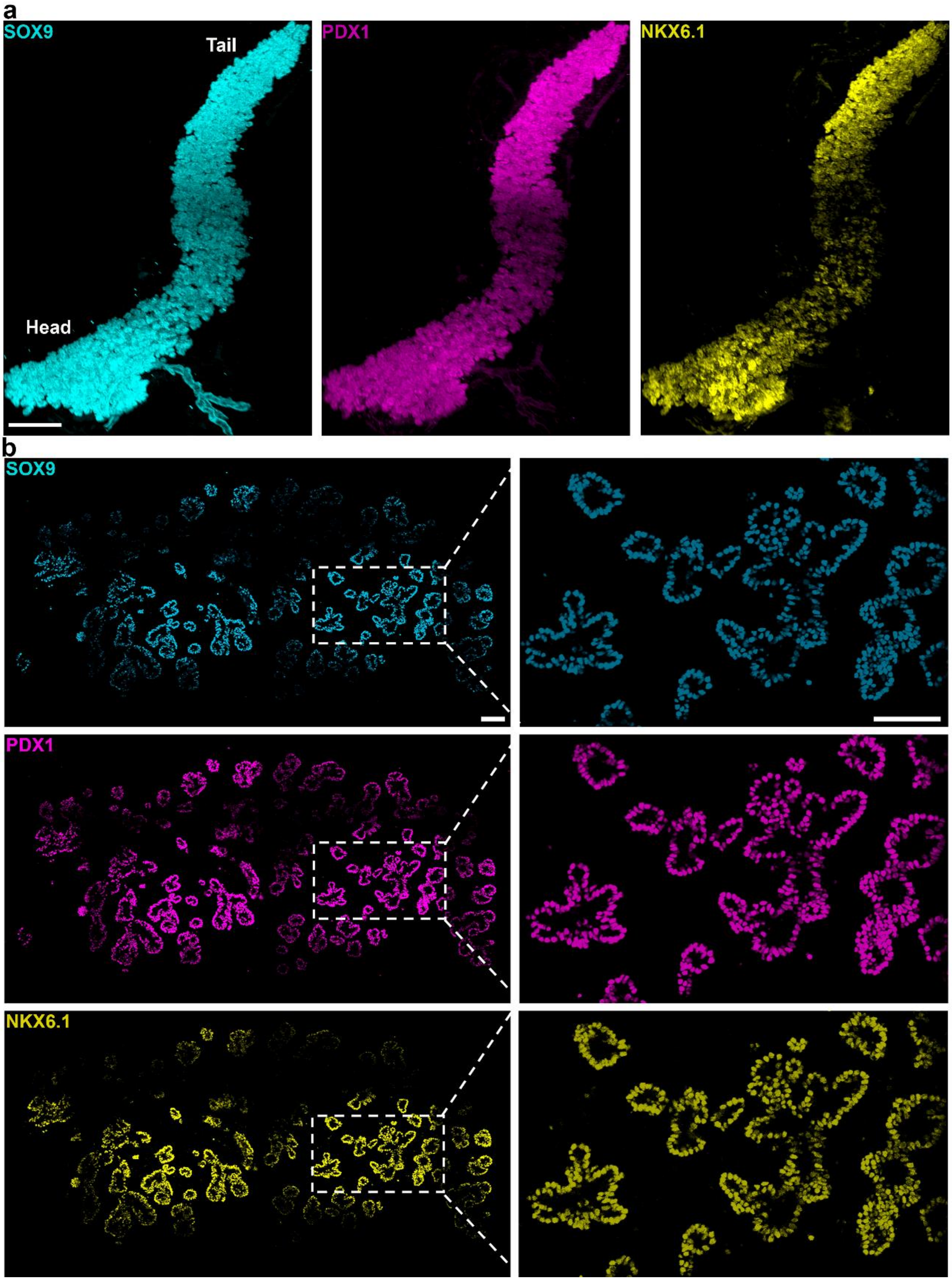

ESM Fig. 1. Expression pattern of SOX9, PDX1 and NKX6.1 in the human fetal pancreatic epithelium. (a) Staining of SOX9 (cyan), PDX1 (magenta) and NKX6.1 (yellow) in the human pancreatic epithelium at PCW8. **(b)** Mosaic images showing the expression pattern of these markers in a human fetal pancreas at PCW8.3 (n=3).

Scale bar: 500µm (a), 100µm (b).

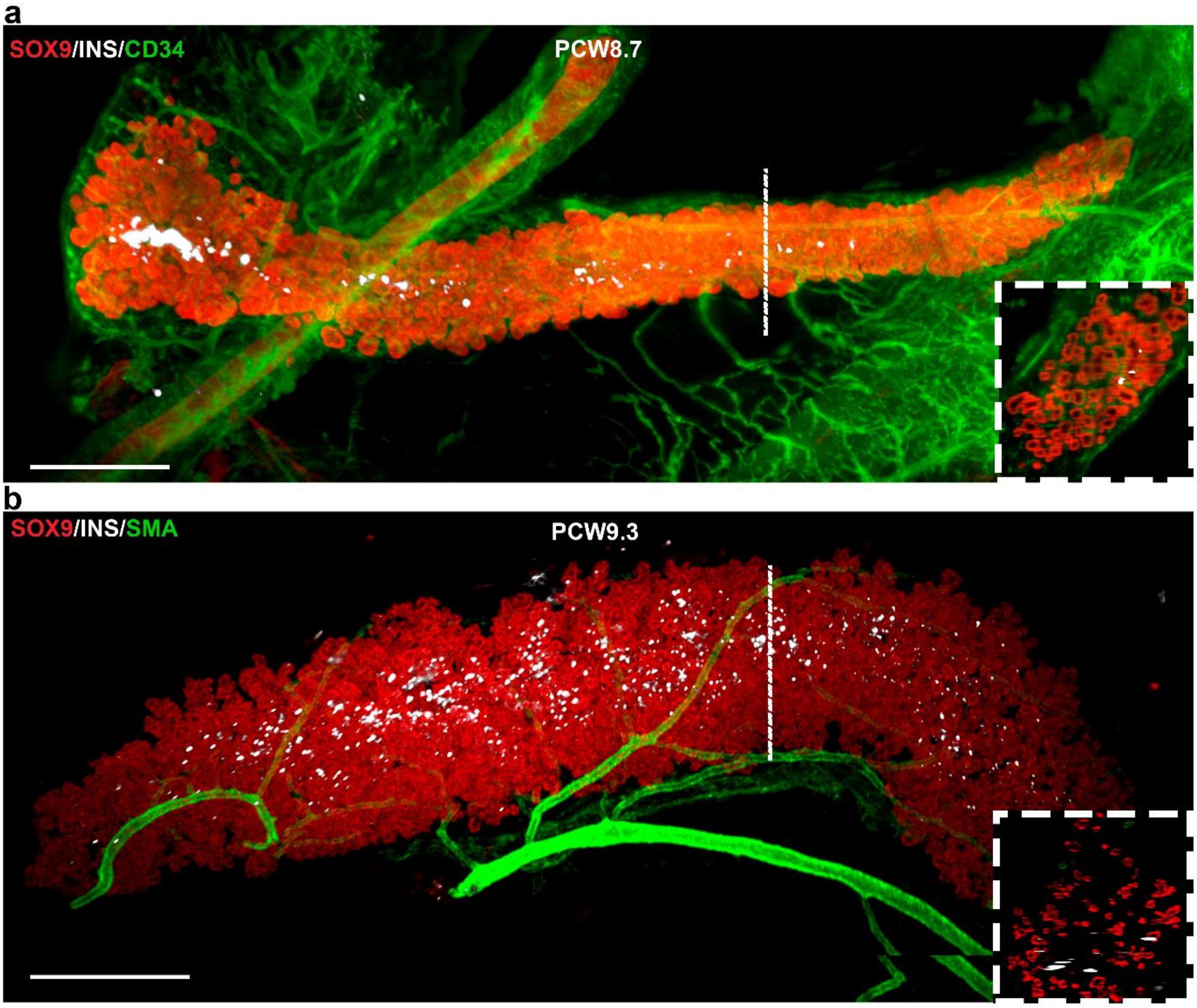

ESM Fig. 2. Vascularisation of the human fetal pancreas. Representative images of (**a)** vascular endothelium (CD34, green) at PCW8.7, and (**b)** arterial 6ascularization (SMA, green) in the human fetal pancreas at PCW9.3 (SOX9 in red, INS in white). Insets: 2D slices of the 3D images in (a, b) (n=3). Slashed lines represent the position of the insets in the 3D image.

Scale bar: 500µm (a and b).

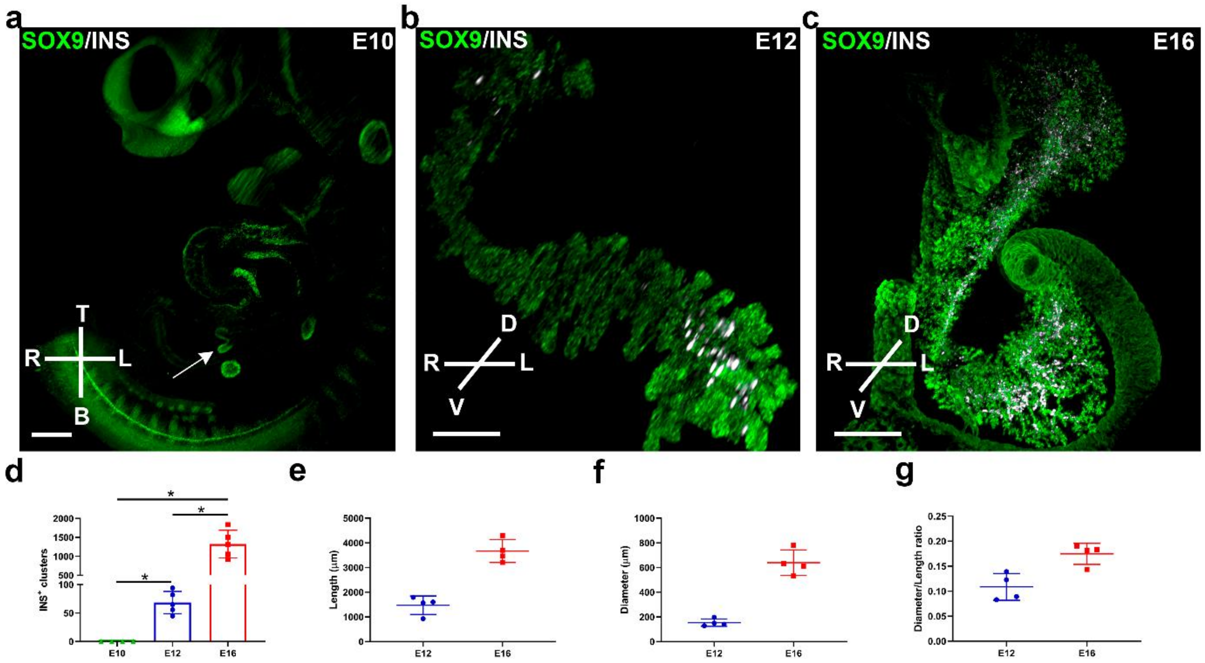

ESM Fig. 3. 3D analysis of pancreatic growth and beta cell development in mice. (**a-c)** Growth of the mouse fetal pancreas from E10 to E16. SOX9 in green and INS in white (n=4). White arrow depicts the pancreatic primordium. (**d-g)** Length (right-left axis), diameter (ventral-dorsal axis), diameter to length ratio measurements and INS$^+$ clusters number in 3D images of murine fetal pancreases at E10, E12 and E16. *P value < 0.05, Mann-Whitney test (n=4).

Scale bar: 500µm (a-c).

Abbreviations: R, right; L, left; V, ventral; D, dorsal; T: Top; B: Bottom.

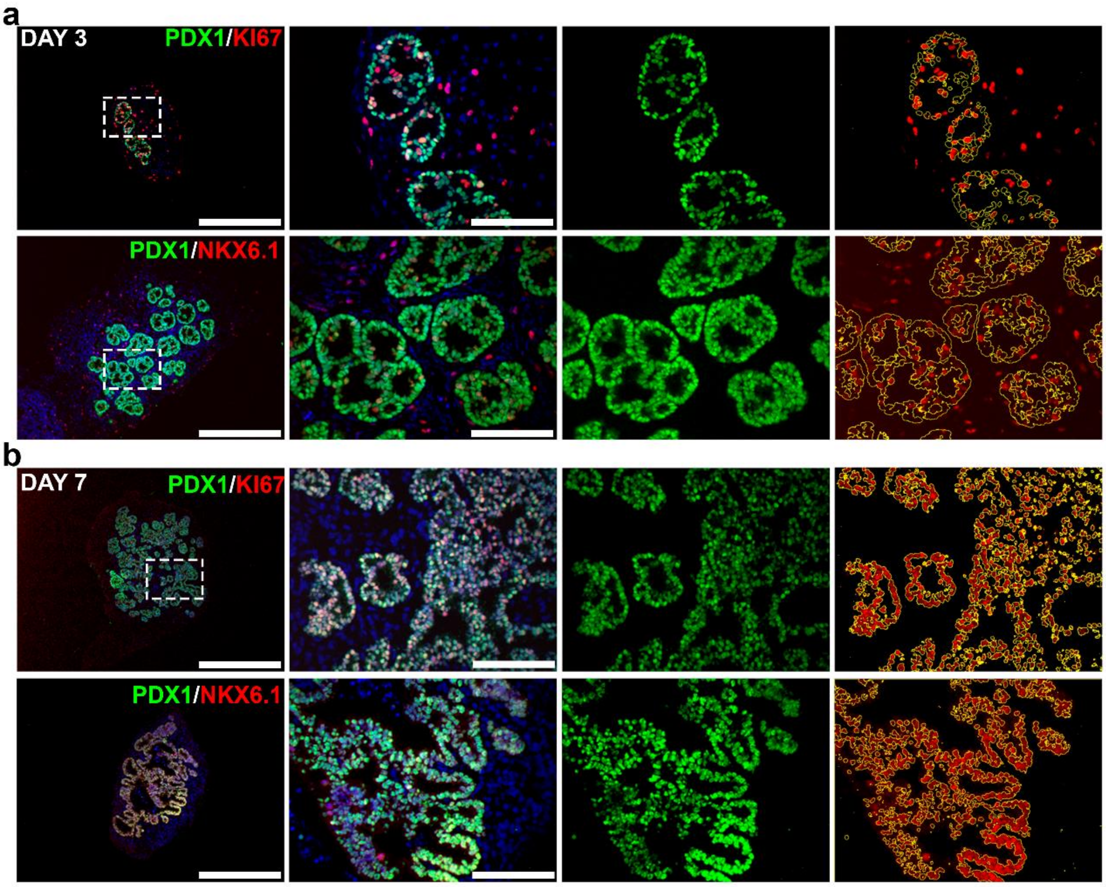

ESM Fig. 4. Determination of multipotency and proliferation of progenitors in cultured human fetal pancreas. (a) Representative staining for PDX1 (green) and KI67 (red) on sections of human fetal pancreas cultured until days 3 or 7 (n=6). (**b)** Representative staining for PDX1 (in green) and the pancreatic multipotency marker NKX6.1 (in red) on sections of human fetal pancreas cultured until days 3 or 7. Yellow overlay indicates the shape of the $PDX1^+$ epithelium (n=6).

ESM Video 1, related to ESM Fig. 1 Expression pattern of SOX9, PDX1 and NKX6.1 in the human fetal pancreatic epithelium. Staining of SOX9 (cyan), PDX1 (magenta) and NKX6.1 (yellow) in the human pancreatic epithelium at PCW8

ESM Video 2, related to Fig. 1 Anatomical location of the pancreas in the human embryo. Human embryo at PCW7 stained with SOX9 and TH in white. The pancreas is highlighted in magenta.

ESM Video 3, related to Fig. 1 Detection of ventral and dorsal buds with light-sheet fluorescence microscopy in human embryonic pancreas at PCW5.7. SOX9 in white

ESM Video 4, related to Fig. 2 Temporal location of the first INS^+ cells in the human embryonic pancreas. Pancreas at PCW5.7 stained with SOX9 in green and INS in white

ESM Video 5, related to Fig. 2 Detection of extra-pancreatic INS^+ cells in the human embryonic gut with light-sheet fluorescence microscopy. Pancreas at PCW6.3 stained with SOX9 in green and INS in white

ESM Video 6, related to Fig. 2 Spatial location of INS^+ clusters in the human fetal pancreas. Pancreas at PCW8 stained with SOX9 in green and INS in white
